# A Class II KNOX Gene, *KNAT7-1*, Regulates Physical Seed Dormancy in Mungbean [*Vigna radiata* (L.) Wilczek]

**DOI:** 10.3389/fpls.2022.852373

**Published:** 2022-03-15

**Authors:** Kularb Laosatit, Kitiya Amkul, Tarika Yimram, Jingbin Chen, Yun Lin, Xingxing Yuan, Lixia Wang, Xin Chen, Prakit Somta

**Affiliations:** ^1^Department of Agronomy, Faculty of Agriculture at Kamphaeng Saen, Kasetsart University, Nakhon Pathom, Thailand; ^2^Center for Advanced Studies for Agriculture and Food, Kasetsart University Institute for Advanced Studies, Kasetsart University, Bangkok, Thailand; ^3^Institute of Industrial Crops, Jiangsu Academy of Agricultural Sciences, Nanjing, China; ^4^Institute of Crop Sciences, Chinese Academy of Agricultural Sciences (CAAS), Beijing, China; ^5^Center for Agricultural Biotechnology, Kasetsart University, Nakhon Pathom, Thailand; ^6^Center of Excellence on Agricultural Biotechnology: (AG-BIO/MHESI), Bangkok, Thailand

**Keywords:** mungbean, seed dormancy, hardseededness, KNOX II, KNAT7

## Abstract

Seed dormancy in wild mungbean (*Vigna radiata* var. *sublobata*) may be useful for the breeding of cultivated mungbean (var. *radiata*) with pre-harvest sprouting resistance. Previous studies have identified two major quantitative trait loci (QTLs) for seed dormancy, *HsA* and *Sdwa5.1.1*+, in wild mungbean that are possibly having the same locus or linked. However, these QTLs have not been confirmed/verified and a molecular basis of seed dormancy in mungbean is not yet known. In this study, we aimed to finely map the *Sdwa5.1.1*+ and identify candidate gene(s) for this locus. Microscopic observations revealed that wild mungbean “ACC41” seeds had a palisade cuticle layer, while cultivated mungbean “Kamphaeng Saen 2” (KPS2) seeds lacked this layer. Fine mapping using an F_2_ population developed from a cross between ACC41 and KPS2 revealed two linked QTLs, *Sdwa5.1.1*+ and *Sdwa5.1.2*+, controlling seed dormancy. The *Sdwa5.1.1*+ was confirmed in an F_2:3_ population derived from the same cross and mapped to a 3.298-Kb region containing only one gene *LOC106767068*, designated as *VrKNAT7-1*, which encodes the transcription factor KNOTTED ARABIDOPSIS THALIANA7 (KNAT7), a class II KNOTTED1-LIKE HOMEOBOX (KNOX II) protein. *VrKNAX7* sequence alignment between ACC41 and KPS2 revealed several polymorphisms in the coding, untranslated, and promoter regions. Quantitative real-time PCR (qRT-PCR) analysis revealed that the expression of *VrKNAT7-1* and *VrCYP86A*, a putative downstream regulation of *VrKNAT7-1*, in the seed coat of ACC41 is statistically much higher than that of KPS2. Altogether, these results indicate that *VrKNAT7-1* controls physical seed dormancy in the wild mungbean ACC41.

## Introduction

Plant domestication, the earliest form of plant breeding, is an evolutionary change of wild plants to domesticated plants that serve human needs, including foods, fibers, medicines, feeds, energies, cosmetics, and ornamentals. Plant domestication is one of the most important events in human history as it transformed human ways of life from the hunting-gathering society to agricultural society approximately 12,000 years ago, which eventually led to present modern and civilized society (Diamond, 1999, 2002; Larson et al., 2014). Processes of plant domestication are associated with forces stemmed from human-mediated selection, both conscious and unconscious selections, under human-manipulated environments. The changes of wild plants include morphological, developmental, and physiological traits. For example, in general, compared with wild plants, domesticated plants possess non-dormant seeds, indehiscent seeds or fruits, larger organs (fruit, seed, leaf, and stem), reduced or no branching, earlier flowering and maturity, tastier, and lower or no toxic and more colorful edible part(s). Those domestication-related traits are called “domestication syndrome” (Hammer, 1984). In the plant domestication, seed dormancy and seed/fruit indehiscence are believed to be the first trait selection as the traits are for advantageous cultivation.

Mungbean [*Vigna radiata* (L.) Wilczek], one of several legume crops of the genus *Vigna*, is an important crop of Asia. The crop is grown into various cropping systems due to its fast growth, early maturity (60–75 days), relatively tolerance to drought, ability to improve soil fertility through atmospheric nitrogen (*N*_2_) fixation in symbiosis with *Rhizobium* species in the soil (Somta and Srinives, 2007). Mungbean seeds are the sources of protein, amino acids, carbohydrates, vitamins, and minerals. Dry seeds of mungbean contain about 20–25% proteins and 60–70% carbohydrates. Whole and split seeds of mungbean are cooked and consumed in a variety of ways. The seeds are an important protein source for people in the cereal-based society, especially in South Asia. The seeds are also used to produce bean sprouts, paste, starches, noodles, protein isolates, and protein concentrates (Nair and Schreinemachers, 2020). Due to its high protein content, mungbean has become an important and a popular source of plant-based proteins. The world production area of mungbean is about 7.2 million ha, of which about 90% is in Asia (Nair and Schreinemachers, 2020). The crop is now gaining increasing popularity in Australia, America, and Africa.

Seed dormancy is a crucial trait for the domestication of cereal and legume crops. Non-dormant seeds are advantageous for uniform and timely cultivation. An understanding of the genetic basis underlying seed dormancy may be useful for exploiting wild genetic resources for the improvement of crops (Imrie et al., 1988; Lawn et al., 1988), especially in the face of climate change. For example, the dormancy may provide the protection of pre-harvest sprouting. In the genus, *Vigna* that comprises 10 domesticated legume crops, there are not many studies reported on quantitative trait locus (QTL) mapping of domestication syndrome, including adzuki bean (Isemura et al., 2007; Kaga et al., 2008), black gram (Somta et al., 2020), cowpea (Kongjaimun et al., 2012; Lo et al., 2018), mungbean (Isemura et al., 2012), moth bean (Yundaeng et al., 2019), and zombi pea (Dachapak et al., 2018; Amkul et al., 2020). One to six QTLs controlled seed dormancy in these legume species. Candidate genes for seed dormancy have only been reported for zombi pea (Amkul et al., 2020). In mungbean, Mendelian genetic analysis showed that seed dormancy in wild mungbean (*V. radiata* var. *sublobata*) accession “Pantnagar” from India, having about 95% dormant seeds is controlled by a single dominant gene, *Hd*_1_ (Singh et al., 1983). Lawn et al. (1988) demonstrated that wild mungbean accession “ACC41” from Australia, with > 95% dormant seeds is likely to be controlled by a single major gene and some modifying factors. QTL mapping in a recombinant inbred line population derived from a cross between commercial mungbean cultivar and ACC41 that was grown under field and glasshouse conditions using restriction fragment length polymorphism (RFLP) markers revealed that the dormancy in ACC41 is controlled by one major and three minor QTLs located on different linkage groups (Humphry et al., 2005). However, only the major QTL, *HsA*, was found in both environments. The *HsA* was located between RFLP markers cgO103 and VrCS364 and explained 23.2% of the dormancy variation. Broad-sense heritability (*H*^2^) was estimated for the dormancy in this population is 90% for both field and glasshouse conditions (Humphry et al., 2005). Similarly, a study using an F_2_ population of a cross between landrace mungbean and wild mungbean accession “JP211874” (99% dormant seeds and from Myanmar) grown in a single environment demonstrated that *H*^2^ for seed dormancy is 99% and the trait is conditioned by two major and two minor QTLs located on different linkage groups (Isemura et al., 2012). Among those QTLs, *Sdwa5.1.1*+ showed the largest effect, explaining 33.7% of the seed dormancy variation. The *Sdwa5.1.1*+ was located on linkage group 1 between simple sequence repeat (SSR) markers cp05137 and CEDG074b. The distance between these markers was large, being 14.2 cM. However, the *Sdwa5.1.1*+ has not been confirmed/validated and a genetic basis of this locus is not yet known. Nonetheless, based on the mungbean linkage map reported by Wang et al. (2016) and BLASTN search revealed that cgO103, VrCS364, cp05137, and CEDG074b are linked (P. Somta, unpublished data), suggesting that *HsA* and *Sdwa5.1.1*+ may be the same locus or different locus but linked. In addition, markers associating with *Sdwa5.1.1*+ also showed a linkage with *Sdg3.1.2*, which is a major QTL conferring seed dormancy in wild adzuki bean (*Vigna angularis* var. *nipponensis*) (Kaga et al., 2008).

In this paper, we report fine mapping of the *Sdwa5.1.1*+ and the identification of candidate genes(s) at this QTL and a novel QTL linked with *Sdwa5.1.1*+. The objectives of this study were to (i) finely map the *Sdwa5.1.1*+ in wild mungbean ACC41 and (ii) identify candidate(s) gene for the *Sdwa5.1.1*+. We demonstrated that a class II KNOTTED1-LIKE HOMEOBOX (KNOX II) gene, *KNAX7-1*, is associated with physical dormancy in ACC41.

## Materials and Methods

### Plant Materials and Population Development

Two mungbean populations, F_2_ and F_2:3_ generations, were used in this study. The F_2_ population comprised 575 individuals developed from the hybridization between ACC41 (female parent) and Kamphaeng Saen 2 (male parent; hereafter called KPS2). ACC41 is a wild mungbean (var. *sublobata*) from Australia and possesses dormant seeds, while KPS2 is a cultivated mungbean (var. *radiata*) from Thailand and possesses non-dormant seeds. This F_2_ population has been previously used for gene mapping of bruchid resistance (Kaewwongwal et al., 2020). The F_2_ plants and parents (10 plants each) were grown under field conditions at Kasetsart University, Kamphaeng Saen Campus, Nakhon Pathom, Thailand from February to May 2018. Mature pods were harvested from each plant and the dry seeds were used for dormancy evaluation. Details of the F_2:3_ generation is described in the section “Confirmation of *Sdwa5.1.1*+ for seed dormancy.”

### Characterization of Seed Dormancy and Morphology

In total, 50 intact seeds of ACC41 and KPS1, and scarified seeds of ACC41 were used for the observation of imbibition and germination test. The seeds were soaked in deionized water, and then imbibition and germination of the seeds were observed and photographed at 0, 4, 6, 8, 10, 12, 14, 16, 18, 20, 22, and 24 h.

Seed coat structure of ACC41 and KPS1 was investigated using microscope following the procedures described by Chai et al. (2016). In brief, mature seeds of both accessions were soaked in sterile water and then fixed with 2.5% glutaraldehyde and 4% paraformaldehyde in a PBS buffer. Then, the seeds were washed with PBS and postfixed in 1% osmium tetroxide, dehydrated in a series of ethanol dilutions, embedded in LR White resin, and polymerized. Cross-sections were cut in the middle of the seed using Leica EM UC7 Ultramicrotome (Leica Microsystems, Germany). Subsequently, semithin sections were stained with 1% toluidine blue O and observed under Nikon Microphot-2 (Nikon Corporation, Japan).

### Evaluation of Seed Dormancy in the F_2_ Population

In total, 50 intact seeds of each F_2_ plant that had been stored at room temperature for 60–80 days after harvest were placed into a hole of a germination tray. Each tray contained 35–40 holes. Deionized water was added into each hole until the seeds were submerged. The tray was then incubated at 25°C with 12-h light and 12-h darkness for 7 days. Deionized water was added to the holes daily, if necessary, to maintain the level of water submergence of the seeds. The number of seeds that not imbibed water was recorded. Percentage of seed dormancy (PSD) of each plant was calculated.

### Development of New DNA Markers for Fine Mapping the *Sdwa5.1.1*+ and Identification of New Quantitative Trait Loci Conferring Seed Dormancy

*Sdwa5.1.1*+ conferring seed dormancy in wild mungbean was previously detected between SSR markers cp05137 and CEDG074b (Isemura et al., 2012). To finely map the *Sdwa5.1.1*+ locus, we determined physical locations of these markers by performing BLASTN search of the primer sequences of cp05137 and CEDG074b against mungbean reference genome (Kang et al., 2014). After the physical genome locations of these two markers were identified, DNA sequence between and around the two locations was searched for SSRs using the software SSRIT (Temnykh et al., 2001). Primers for the SSRs were designed using the software Primer3 (Untergasser et al., 2012).

It has been reported that *Sdg3.1.2* is a major QTL conferring seed dormancy in wild adzuki bean (*Vigna angularis* var. *nipponensis*) (Kaga et al., 2008). SSR markers CEDG214 and CEDG256 associating with the *Sdg3.1.2* (Kaga et al., 2008) have been shown to be linked with the marker cp05137 in the mungbean (Isemura et al., 2012). Existence of QTL homologous to the *Sdg3.1.2* in the wild mungbean ACC41 was investigated. To do so, locations of the CEDG214 and CEDG256 on the mungbean reference genome (Kang et al., 2014) were determined by BLASTN (Altschul et al., 1990). Subsequently, SSRs lying between and around these markers were searched and used to develop SSR markers using the same software described above.

In total, 405 SSR markers were screened for polymorphism between ACC41 and KPS2 (Supplementary Table 1). A marker analysis was carried out as per Yundaeng et al. (2021). In brief, PCR was carried out in a total volume of 10 μl containing 5 ng of DNA template, 1 × *Taq* buffer, 2 mM MgCl_2_, 0.2 mM dNTPs, 1 U *Taq* DNA polymerase, and 2.5 μM each of forward and reverse primers. Amplification was performed at 94°C for 3 min followed by 35 cycles of 94°C for 30 s, 55°C for 30 s, 72°C for 30 s, and 72°C for 10 min. PCR products were electrophoresed on 5% polyacrylamide gel electrophoresis and visualized by silver staining. In total, 24 markers showing polymorphic and unambiguous DNA bands were used to genotype the F_2_ population.

### Linkage Map Construction and Quantitative Trait Loci Analysis in the F_2_ Population

A genetic linkage map was constructed for the F_2_ population using the software QTL IciMapping 4.2 (Meng et al., 2015). The markers were grouped with a logarithm of the odds (LODs) value of 5.0. Orders of the markers on the linkage group were determined by the REcombination Counting and ORDering (RECORD) algorithm (Van Os et al., 2005) and rippled by the Sum of Adjacent Recombination Frequencies (SARF) function (Falk, 1989). Genetic distance in centimorgan unit (cM) between the markers was calculated using the Kosambi mapping function. Location of the QTLs was determined by an inclusive composite interval mapping (ICIM) (Li et al., 2007) using the same software for a linkage analysis. ICIM was performed at every 0.1 cM. Significant LOD score threshold for the QTL was determined by running a 5,000-permutation test at *p* = 0.001.

### Confirmation of *Sdwa5.1.1*+ for Seed Dormancy

Based on the results from the QTL analysis in the F_2_ population that two linked QTLs, *Sdwa5.1.1*+ and *Sdwa5.1.2*+, were identified for seed dormancy, we selected 15 F_2_ plants that were heterozygous at flanking markers of the QTLs *Sdwa5.1.1*+. In total, 10–15 F_3_ seeds from the selected F_2_ plants were grown under field conditions at Kasetsart University, Kamphaeng Saen Campus, Nakhon Pathom, Thailand from February to May 2020. Finally, 112 F_3_ plants were used to confirm the *Sdwa5.1.1*+. Five markers were selected and used for confirmation. DNA extraction was carried out as per Lodhi et al. (1994). A marker analysis, seed dormancy evaluation analysis, and QTL analysis were the same as described above.

### Sequencing of Candidate Gene, *VrKNAT7*-*1*

Once the QTLs for seed dormancy were identified, mungbean reference genome (Kang et al., 2014) was inspected to identify candidate gene(s). Annotated genes locating between markers flanking each QTL were considered as candidate gene(s) for the dormancy. Only candidate gene, *LOC106767068* (*VrKNAT7-1*), at the *Sdwa5.1.1*+ was sequenced because its location was confirmed. Coding sequence (CDS), 5′-untranslated region (5′UTR), 3′-untranslated region (3′UTR), and 884-bp upstream sequence of the gene were amplified from the genomic DNA of ACC41 and KPS2 using the primers listed in Supplementary Table 1. PCR was carried out in a total volume of 10 μl containing 5 ng of DNA template, 1 × *Taq* buffer, 2 mM MgCl_2_, 0.2 mM dNTPs, 1 U KOD-Plus-Neo DNA polymerase (TOYOBO, China), and 0.5 μM each of forward and reward primers. PCR was performed in SimpliAmp thermal cycler (Applied Biosystems, United States) programmed as follows: 94°C for 2 min followed by 35 cycles of 94°C for 30 s, 55°C for 30 s, 72°C for 1 min, and 72°C for 10 min. PCR products were run on 1.5% agarose gel electrophoresis to confirm the single DNA fragment was amplified. The fragments were Sanger sequenced using ABI 3730xl DNA Analyzer (Applied Biosystems, United States) by Tsingke (Beijing, China). The sequences of KPS2, ACC41, and reference sequence (VC1973A; Kang et al., 2014) were aligned to identify polymorphism(s) using Clustal Omega (Sievers et al., 2011). The CDSs of ACC41 and KPS2 were translated into protein sequences and aligned to find amino acid polymorphism.

### Expression Analysis of the Candidate Gene

ACC41 and KPS2 were grown in a crossing block. Total RNA was extracted from flowers, pods, the seed coat, and seeds (cotyledons and embryos) of both accessions following the protocol described by Laksana and Chanprame (2015). The RNA was converted into complementary DNA (cDNA) using the RevertAid H Minus First Strand cDNA Synthesis kit (Thermo Scientific, United States). cDNA concentration was quantified by ND-1000 Spectrophotometer (NanoDrop Technologies, Inc., United States). The cDNA was subjected to a gene expression analysis by a quantitative real-time PCR (qRT-PCR). Primers for qRT-PCR of the candidate gene *VrKNAT7-1* (*LOC106767068*) and reference gene *VrACTIN* (*LOC106770112*) were designed using the Primer 3 (Supplementary Table 1). qRT-PCR was performed using ViiA 7 Real-Time PCR System (Applied Biosystems, United States). Three biological and technical replicates were conducted for ACC41 and KPS2. Reaction mixtures contained water, 1 × Master mix of Fast SYBR™ Green Master Mix (Thermo Fisher Scientific, United States), 5 μM of forward primer, 5 μM of reverse primer, and 50 ng cDNA. Thermocycle conditions included initial denaturation at 95°C for 20 s, followed by 40 cycles at 95°C for 3 s and 60°C for 30 s. After 40 cycles, a melting curve was generated by slowly increasing (0.5°C per 1 s) the temperature from 60 to 95°C, while the fluorescence was measured. Fluorescent data were acquired during each extension phase. Expression levels of the *VrKNAT7-1* were calculated based on the ΔC_T_ method by using *ACTIN* as the reference (Livak and Schmittgen, 2001). Statistical differences in the gene expression level between ACC41 and KPS2 were tested by a *t*-test at 1% probability using R program version 2.10.0.

Because *VrKNAT7-1* is an ortholog of *Medicago truncatula KNOX4* (*MtKNOX4; Medtr5g011070*) in which that latter is a transcription factor and has been reported to regulate the expression of *CYP86A* (*MtCYP86A; Medtr8g030590*) that is involved in cuticle biosynthesis (Chai et al., 2016), we determined whether the expression of *VrCYP86A* (*LOC106774045*) which is an ortholog of *MtCYP86A* in ACC41 and KPS2 is different. Expression analysis of *VrCYP86A* was the same as that of the *VrKNAT7-1*.

### Phylogenetic Analysis of KNOXs

VrKNAT7-1 (XP_014507374.1) and VrKNAT7-2 (XP_014521195.1) together with KNOX protein sequences of *Arabidopsis thaliana* [AtKNAT1 (AT4G08150.1), AtKNAT2 (AT1G70510.1), AtKNAT3 (AT5G25220.1), AtKNAT4 (AT5G11060.1), AtKNAT5 (AT4G32040.1), AtKNAT6 (AT1G23380.1), AtKNAT7 (AT1G62990.1), and AtKNATM (AT1G14760.1)] and of *M. truncatula* (MtKNOX1 (Medtr2g024390.1), MtKNOX2 (Medtr1g017080.1), MtKNOX3 (Medtr1g012960.1), MtKNOX4 (Medtr5g011070.1), MtKNOX5 (Medtr3g106400.1), MtKNOX6 (Medtr5g085860.1), MtKNOX7 (Medtr5g033720.1), MtKNOX8 (Medtr1g084060.1), MtKNOX9 (Medtr4g116545.1), MtKNOX10 (Medtr2g461240.1), MtFCL1 (Medtr6g071190.1), and MtFCL2 (Medtr1g032750.1) were used for a phylogenetic analysis. The KNOX sequences of mungbean were from the GenBank database, whereas those of *A. thaliana* and *M. truncatula* were from TAIR^1^ and Phytozome13^2^ databases, respectively. The phylogenetic analysis was carried out by the software using Phylogeny.fr (Dereeper et al., 2008) in which the sequences were aligned by MUSCLE 3.7 and constructed into a tree by maximum likelihood with 1,000 bootstraps.

## Results

### Seed Morphology and Dormancy of ACC41 and KPS2

**FIGURE 1:**
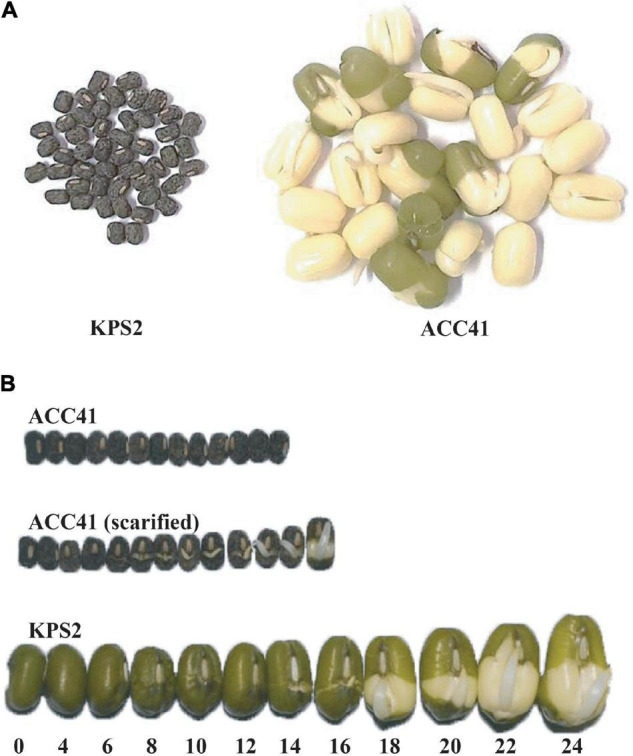
Seed germination test of the wild mungbean ACC41 and the cultivated mungbean Kamphaeng Saen 2 (KPS2). The germination of intact seeds of ACC41 and KPS2 at 7 days after the test **(A)**. The germination of intact seeds of KPS2 and ACC41 and scarified seeds of ACC41 at different time series (0–24 h) **(B)**.

The wild mungbean ACC41 and the cultivated mungbean KPS2 showed a clear difference in seed dormancy (Figure 1A). In the germination test (Figure 1B), nearly all the intact seeds of ACC41 were static throughout the course of the germination test, 7 days. However, when the seeds of ACC41 were scarified and subjected to germination test, all the seeds imbibed water rapidly and germinated at 12 h (Figure 1B). In contrast, all intact seeds of KPS2 started imbibing water at 4 h as shown by swelling of the seeds (Figure 1B). The seed coat of KPS2 became wrinkle at 8 h and cracked at 14 h. The seeds eventually germinated at 16 h as shown by a protrusion of the radicle. The percentage of dormant seeds (PDSs) in ACC41 was 96.0%, while that in KPS2 was 0%.

**FIGURE 2:**
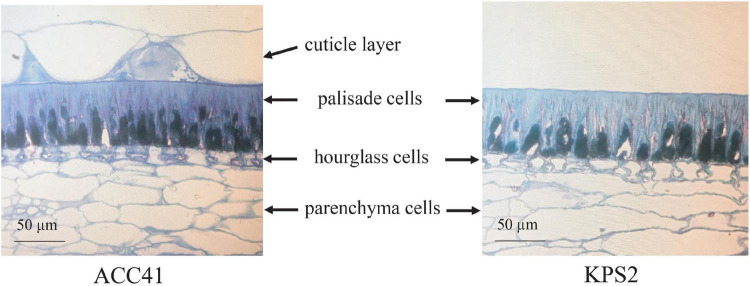
Transverse sections of the seed coat at the maturation stage of the cultivated mungbean of wild mungbean ACC41 and cultivated mungbean KPS2, stained with 0.05% toluidine blue O.

Microscopic observations of cross-sections of the intact seeds of ACC41 and KPS2 revealed that the seed coat of the two accessions possessed similar parenchyma, hourglass, and palisade cells (Figure 2). Nonetheless, the seed coat of ACC41 had a palisade cuticle layer, whereas that of KPS2 lacked the palisade cuticle layer (Figure 2). In ACC41, the thickness of a cuticle layer was almost the same as that of a palisade cell.

### Fine Mapping for the *Sdwa5.1.1*+ and Identification of New QTLs for Seed Dormancy

**FIGURE 3:**
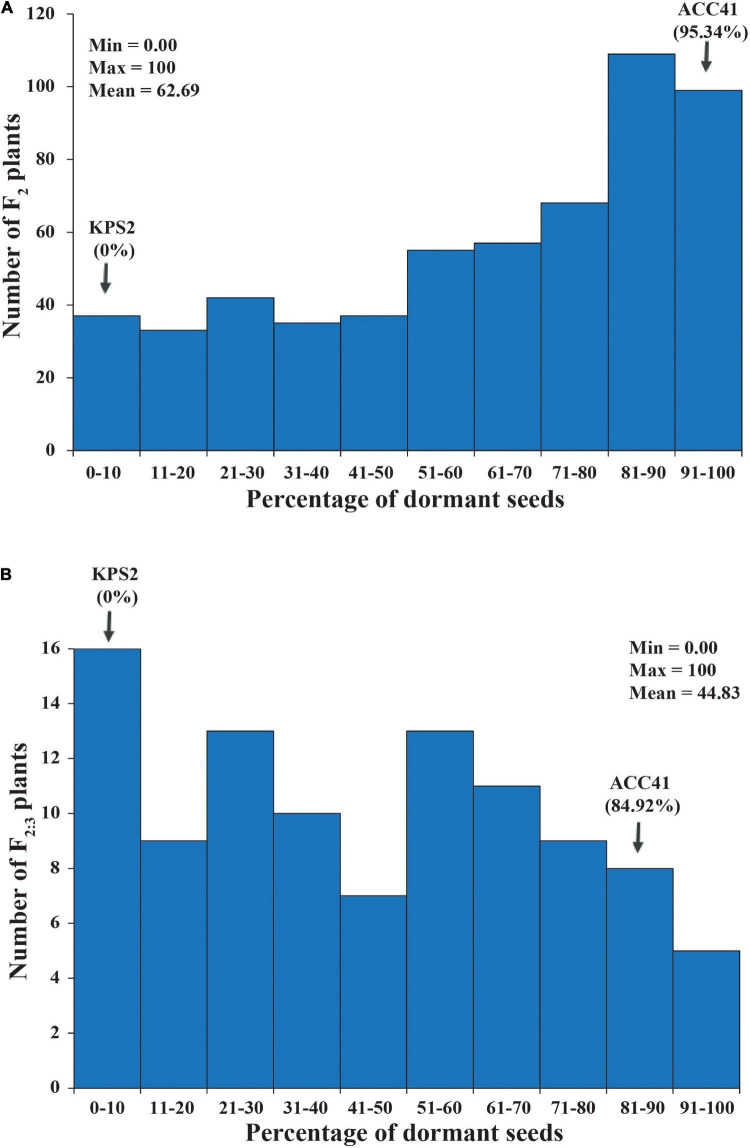
Frequency distribution of the percentage of dormant seeds (PDSs) in F_2_ population of 573 individuals **(A)** and F_2:3_ population of 100 individuals **(B)** derived from the cross between KPS2 and ACC41.

In the F_2_ population of KPS2 × ACC41, PDSs varied between 0% and 100% with a mean of 62.68%. ACC41 had a PDS of 95.34%, while KPS2 had a PDS of 0%. The PDS of the F_2_ population showed continuous distribution, but skewed into the direction of ACC41 (Figure 3A). Broad-sense *H*^2^ estimated for this population was high, being 97.01%.

The BLASTN search revealed that physical locations of the markers cp05137 and CEDG074b that delimited the QTL *Sdwa5.1.1*+ in the study of Isemura et al. (2012) on the mungbean reference genome were at the positions 31,187,230 and 31,822,876 Mbp, respectively, on chromosome 7. Thus, the two markers were 635.65 Kb apart. In addition, to identify a new QTL for seed dormancy that may linked with the *Sdwa5.1.1*+, locations of the CEDG214 and CEDG256 associated with the *Sdg3.1.2* controlling seed dormancy in adzuki bean on the mungbean reference genome were determined. The results showed that they were on the chromosome 7 at the positions 26,548,328 and 27,596,788 in that order. In total, 361 newly developed SSR and Indel markers together with 10 SSR markers reported for seed dormancy QTLs in mungbean and adzuki bean were screened for polymorphism between KPS2 and ACC41. In total, 23 markers showing unambiguous and polymorphic DNA bands were used to analyze the F_2_ population. A linkage group constructed from these markers spanned 21.69 cM in length.

**FIGURE 4:**
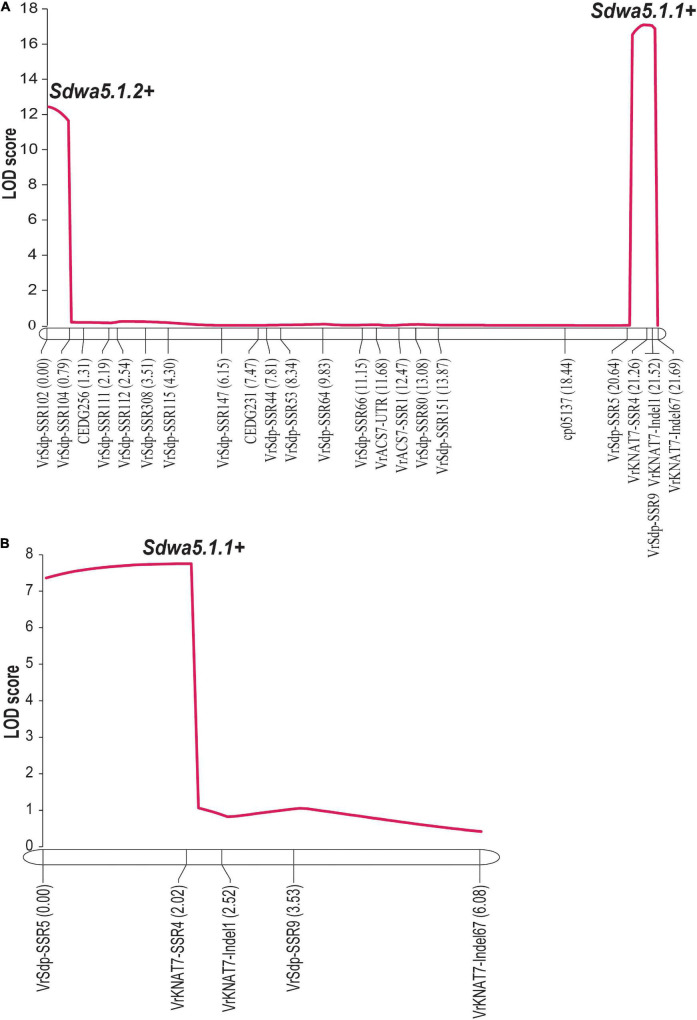
Logarithm of the odd (LOD) graphs of quantitative trait loci (QTLs) for seed dormancy detected on linkage group 1 of F_2_ population of 573 individuals **(A)** and F_2:3_ population of 100 individuals **(B)** derived from the cross between KPS2 and ACC41. Dotted line parallel to *x*-axis represents LOD threshold for the QTLs.

An inclusive composite interval mapping analysis in the F_2_ population revealed two linked QTLs controlling seed dormancy, designated as *Sdwa5.1.1*+ and *Sdwa5.1.2*+ (Figure 4A and Table 1). *Sdwa5.1.1*+ was located between the markers VrSdp-SSR5 and VrKNAT7-SSR4, while *Sdwa5.1.2*+ was located between the markers VrSdp-SSR102 and VrSdp-SSR104. These QTLs were 21.1 cM apart. *Sdwa5.1.1*+ accounted for 17.53% of the dormancy variation in the population and possessed additive effect of −9.12 and dominant effect of 11.55. *Sdwa5.1.2*+ explained 12.27% of the dormancy variation in the population and had additive and dominant effects of −10.91 and 2.27, respectively. At both QTLs, allele(s) from the wild mungbean ACC41 increased the PDS.

**TABLE 1:** Locations and effects of quantitative trait loci on linkage group 2 controlling seed dormancy detected in the F_2_ population of 575 individuals derived from hybridization between cultivated mungbean Kamphaeng Saen 2 (KPS2) and wild mungbean ACC41.

QTL name	Position on linkage group (cM)	LOD score	Interval markers	Phenotypic variance explained (%)	Additive effect	Dominant effect
*Sdwa5.1.2*+	0.00	12.43	VrSdp-SSR102–VrSdp-SSR104	12.27	−10.90	2.27
*Sdwa5.1.1*+	21.10	17.10	VrSdp-SSR5–VrKNAT7-SSR4	17.53	−9.12	11.55

The QTL *Sdwa5.1.1*+ identified in the F_2_ population was confirmed using an F_3_ population of 112 individuals. PDS in the F_2:3_ population ranged from 0 to 100% with a mean of 62.68%. ACC41 had a PDS of 95.34%, while KPS2 had a PDS of 0%. The PDS showed continuous distribution and skewed toward KPS2 (Figure 3B). The *H*^2^ value estimated for the F_2:3_ population was nearly the same with that of the F_2_ population, 97.08%. The ICIM analysis in the F_2:3_ population confirmed that the *Sdwa5.1.1*+ located between the markers VrSdp-SSR5 and VrKNAT7-SSR4 (Figure 4B and Supplementary Table 2). The *Sdwa5.1.1*+ accounted for 31.75% of the dormancy variation in the F_2:3_A population and possessed an additive effect of −18.42 and a dominant effect of 11.85. Again, the allele(s) from ACC41 increased the dormancy.

### Identification of Candidate Genes for the *Sdwa5.1.1*+ and *Sdwa5.1.2*+

**FIGURE 5:**
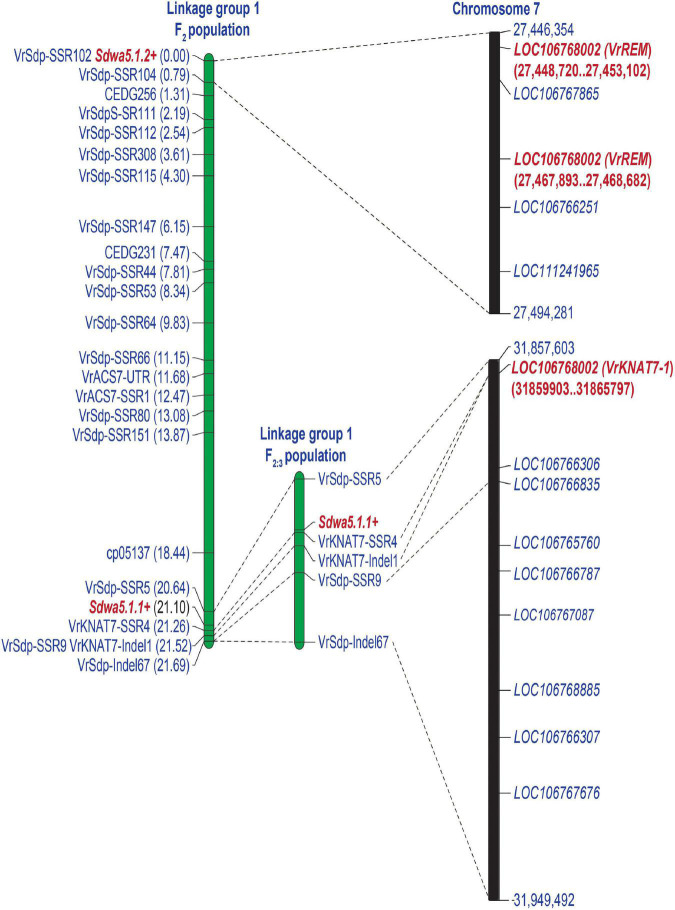
A comparative map illustrating the position of the QTLs *Sda5.1.1*+ and *Sdwa5.1.2*+ controlling seed dormancy on the reference genome of mungbean (VC1973A). Candidate genes at the *Sdwa5.1.1*+ and *Sdwa5.1.2*+ are highlighted in red and bold.

The *Sdwa5.1.1*+ was identified and confirmed in two populations. Based on the mungbean reference genome, the physical region of the marker interval VrSdp-SSR5 and VrKNAT7-SSR4 covering the *Sdwa5.1.1*+ was only 3,298 bp (Figure 5). There was only one annotated gene between the two markers, *LOC106767068*. *LOC106767068* encodes a transcription factor KNOTTED ARABIDOPSIS THALIANA 7 (KNAT7). We designated *LOC106767068* as *VrKNAT7-1* and considered it as the candidate gene for the *Sdwa5.1.1*+. It is noteworthy that the marker VrKNAT7-SSR4 was developed from *VrKNAT7-1*.

The *Sdwa5.1.2*+ was identified in only one population. Physical location of the marker interval VrSdp-SSR102 and VrSdp-SSR104 delimited the *Sdwa5.1.2*+ was 47,927 bp (Figure 5). There were 5 annotated genes in this region, including *LOC106768002, LOC106767865, LOC106767275, LOC106766251*, and *LOC111241965* (Figure 5 and Supplementary Table 3). Among these genes, *LOC106768002* encoding B3 domain-containing protein, with homology to Os01g0234100 from rice, and *LOC106767275* encoding calmodulin-like (CML) protein 1 were considered as the candidate gene for the *Sdwa5.1.2*+. BLASTN search against the TAIR database revealed that *LOC106768002* showed the best hit with *Reproductive Meristem 1* (*At3g19184*), so we designated *LOC106768002* as *VrREM1*. In case of *LOC106767275*, we designated it as *VrCML1*.

### Nucleotide Polymorphisms in *VrKNAT7-1*

**FIGURE 6:**
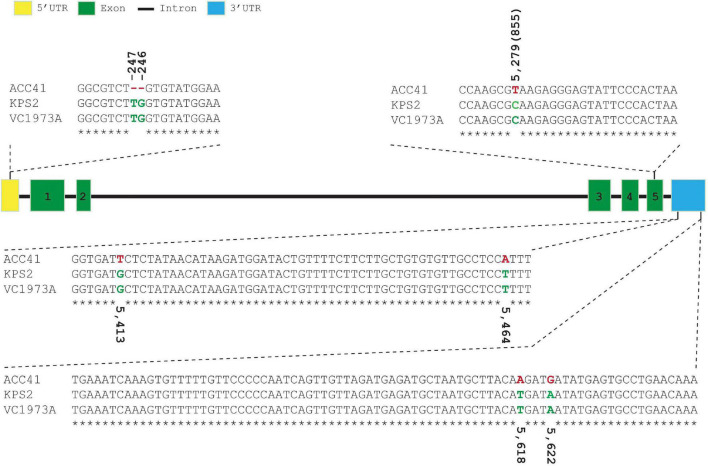
Sequence alignment of 5′-untranslated region, coding sequencing, and 3′-untranslated region of the *LOC106767068* (*VrKNAT7-1*) among ACC41, KPS2 and mungbean reference genome sequence (VC1973A). The number in parenthesis upper/under nucleotide(s) indicates its position from the first nucleotide of the start codon.

*VrKNAT7-1* of ACC41 and KPS2 were sequenced and compared with the reference sequence (VC1973A). *VrKNAT7-1* sequence alignment revealed no polymorphism between the cultivated mungbeans KPS2 and VC1973A, but showed several polymorphisms between ACC41 and KPS2 (Figure 6); one single-nucleotide polymorphism (SNP) in the CDS, one SNP and a 2-bp insertion/deletion (Indel) in the 5′UTR, and four SNPs in the 3′UTR. In addition, an alignment of the *VrKNAT7-1* upstream sequences showed several SNPs and InDels between ACC41 and KPS2 (Supplementary Figure 1). Nonetheless, the SNP in the CDS was a synonymous mutation (Supplementary Figures 2, 3).

### Gene Expression Analysis

**FIGURE 7:**
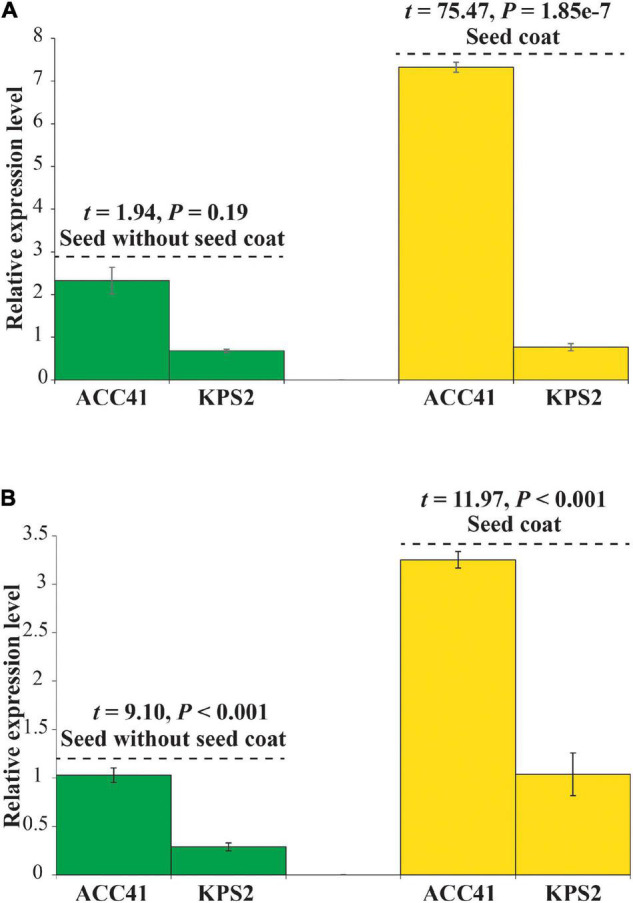
Relative gene expression level of *VrKNAT7-1* (*LOC106767068*) **(A)** and *VrCYP86A* (*LOC106774045*) **(B)** in a seed without seed coat and with the seed coat of ACC41 and KPS2.

The expression of the *VrKNAT7-1* and *VrCYP86A* in a seed without the seed coat and with the seed coat of ACC41 and KPS2 was determined by qRT-PCR. In case of *VrKNAT7-1*, the analysis revealed no significant difference in the seed without seed coat between ACC41 and KPS2, but showed a statistical difference in the seed coat between the two mungbeans (Figure 7A). The expression in ACC41 was about 10-fold higher than KPS2. For *VrCYP86A*, the analysis revealed that the expression in ACC41 was significantly higher than that in KPS2 in both seeds without the seed coat and with the seed coat (Figure 7B).

### Phylogenetic Analysis of VrKNAT7 Proteins

**FIGURE 8:**
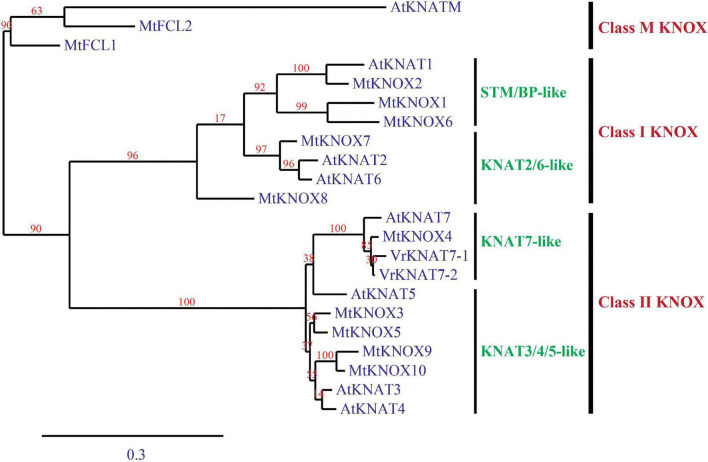
A phylogenic tree of *Vigna radiata* KNAT7-1 and KNAT7-2 proteins, and *Arabidopsis thaliana* and *Medicago truncatula* KNOX proteins. The tree is constructed by a maximum-likelihood method.

Mungbean contained two KNAT7 proteins, VrKNAT7-1 and VrKNAT7-2. A phylogenetic analysis revealed three major clusters (classes) of KNOX proteins, namely class M KNOX, class I KNOX, and class II KNOX (Figure 8). The analysis also confirmed the close relationship between the VrKNAT7-1 and VrKNAT7-2 and demonstrated that both of them were sub-clustered with other KNAT7-like proteins, MtKNOX4 and AtKNAT7 (Figure 8). These KNAT7-like proteins were clustered with the KNOX sub-cluster KNAT3/4/5-like proteins, forming the class II KNOX proteins.

## Discussion

A decrease in or a loss of seed dormancy is an important biological mechanism of crop domestication that enables seeds to germinate uniformly and timely. Although seed dormancy is problematic for crop cultivation, it may be useful for preventing pre-harvest sprouting of seed yield. In general, cultivated mungbeans are suppressed for seed dormancy although a few mungbean germplasms exhibits fresh-seed dormancy for a short period of time (4 days) (Lamichaney et al., 2018). In this study, the *H*^2^ estimated for the seed dormancy in the F_2_ and F_3_ populations was *H*^2^ (90–91%). This result agrees with those from the previous studies that seed dormancy in mungbean is a highly heritable trait with a *H*^2^ value of 95–99% (Lawn et al., 1988; Humphry et al., 2005; Isemura et al., 2012).

Seed dormancy in wild mungbean is controlled by one or two major QTLs together with 2–3 minor QTLs (Humphry et al., 2005; Isemura et al., 2012). Isemura et al. (2012) reported that *Sdwa5.1.1*+ is the major QTL controlling seed dormancy in wild mungbean. However, this QTL was identified in only one environment and has not been confirmed/validated. In this study, we validated the major QTL *Sdwa5.1.1*+ using wild mungbean accession ACC41 as the source of seed dormancy. The *Sdwa5.1.1*+ was previously mapped between the markers cp05137 and CEDG074b (Isemura et al., 2012). Based on the mungbean reference genome sequence (Kang et al., 2014), these flanking markers were located on chromosome 7 at the positions 31,187,230 and 31,822,876, respectively. However, our high-resolution mapping results in both F_2_ and F_3_ populations consistently demonstrated that the *Sdwa5.1.1*+ resides between the markers VrSpd-SSR5 and VrKNAT7-SSR4 (Figure 4), which corresponded to the positions 31,857,603 and 31,860,901 of chromosome 7, respectively (Figure 5). Thus, the region of the *Sdwa5.1.1*+ mapped in our study is different from that reported by Isemura et al. (2012), albeit the two QTL regions are only about 34.7 Kb apart. The contrasting results are possibly due to different sizes of mapping populations used in two studies. The population used in our study comprised 575 individuals, which is about 2.3-fold larger than the one used by Isemura et al. (2012). Larger mapping population size provides a better accuracy of the QTL location (Tanksley, 1993; Charmet, 2000).

Physical seed dormancy, also called hard-seededness, is an adaptive trait for the survival of wild progenitors of seed crops. This type of dormancy is caused by the existence of a water-impermeable layer in the seed coat (Finch-Savage and Leubner-Metzger, 2006). In the genus *Vigna* subgenus *Ceratotrapis*, to which mungbean belongs, seeds of species in this taxon imbibe water through the lens (strophiole) near the hilum (Gopinathan and Babu, 1985; Kikuchi et al., 2006). In this study, intact seeds of ACC41 and KPS2 showed a contrasting degree of dormancy (Figure 1A), while scarified seeds of ACC41 germinated rapidly (Figure 1B). Microscopic observation of seeds clearly showed that the seed cuticle layer was present in ACC41, but absent in KPS2 (Figure 2). These results indicated that the presence of cuticle/cutin causes the physical dormancy in the wild mungbean ACC41. *VrKNAT7-1* was identified as the only candidate gene for seed dormancy at the locus *Sdwa5.1.1*+ (Figure 5). *VrKNAT7-1* encodes a transcription factor homeobox protein HD1 (KNAT7), a KNOX II protein. In *M. truncatula* L., a model legume species, genetic, and molecular analyses revealed that *MtKNOX4*, a class II KNOX gene, controls seed physical dormancy (Chai et al., 2016). NCBI BLASTP search of MtKNOX4 protein against the reference protein database of mungbean showed that the MtKNOX4 was best matched with XP_014521195.1 (95% query coverage, *E*-value = 0.0, and 89.04% identity), followed by VrKNAT7-1 (96% query coverage, *E*-value = 9^e–169^, and 80.95% identity). XP_014521195.1 protein is encoded by *LOC106777892*, designated *VrKNAT7-2*, localizing on mungbean chromosome 11. A phylogenetic analysis further supports a close relationship among VrKNAT7-1, VrKNAT7-2, MtKNOX4, and MtKNAT7 (Figure 8). These results demonstrated that there are two *VrKNAT7* genes in mungbean and both *VrKNAT7-1* and *VrKNAT7-2* are orthologs of *MtKNOX4*. A mutation causing the loss of function of the *MtKNOX4* resulted in the reduction of hydroxylated fatty acids in the seed coat (especially18:2 ω-hydroxy fatty acid), a group of lipid polyester monomer composition of seed cutin, that alters cuticle layer permeability, and thus the physical dormancy of *M. truncatula* (Chai et al., 2016). *MtKNOX4* controls seed dormancy by regulating the expression of *MtCYP86A* gene (Chai et al., 2016) that plays an important role in the cuticle biosynthesis. In *Arabidopsis*, CYP86A2, a cytochrome P450 monooxygenase catalyzing fatty acid oxidation, is required for the biosynthesis of cutin and cuticle development (Xiao et al., 2004). In *M. truncatula*, MtKNOX4 protein directly binds to the promoter of *MtCYP86A*, and thus *MtKNOX4* is believed to regulate cuticle biosynthesis pathway in the seed coat (Chai et al., 2016). In our study, the expressions of *VrKNAT7-1* and *VrCYP86A* in the seed coat of ACC41 and KPS2 collected at a yellow-pod stage revealed that expressions of these genes in ACC41 were statistically much higher than those in KPS2 (Figure 7). The different expression of *VrKNAT7-1* between ACC41 and KPS2 is very likely to be caused by nucleotide polymorphisms in the upstream sequence, 5′ UTR and 3′ UTR of this gene (Figure 6 and Supplementary Figure 1). Nonetheless, these results demonstrated that the *VrKNAT7-1* regulate cutin biosynthesis during seed coat development of the wild mungbean by controlling the expression level of the *VrCYP86A*, and that a common mechanism of physical seed dormancy exists between the different wild species of the genus *Vigna* and *Medicago*.

Apart from physical dormancy, physiological dormancy widely exists in seed-plant species. In fact, the majority of seeds exhibit physiological dormancy in which germination is prevented by using germination-inhibiting hormones, including abscisic acid (ABA) and gibberellins (GAs) (Penfield, 2017). In the previous studies, *Sdwa5.1.1*+ (*HsA*) was identified as a single QTL on the LG1 controlling seed dormancy (Humphry et al., 2005; Isemura et al., 2012). Nonetheless, our QTL analysis revealed that apart from *Sdwa5.1.1*+, *Sdwa5.1.2*+ was also detected on LG1 for the trait (Figure 4). The genetic effects of *Sdwa5.1.1*+ and *Sdwa5.1.2*+ are comparable. The environmental factor(s) may explain contrasting results between the present study and the previous ones regarding the *Sdwa5.1.2*+. Although we did not confirm the *Sdwa5.1.2*+, based on physical location of *Sdwa5.1.2*+ and function of the genes in this QTL region, we considered *VrREM1* and *VrCML1* as candidate genes at this QTL (Figure 6 and Supplementary Table 2). As *VrREM1* encodes a B3 domain-containing protein, with a homolog to Os01g0234100 from rice, it is likely that *VrREM1* is a transcription factor. B3 transcription factors regulate expressions of seed genes during embryogenesis, maturation, dormancy, and germination [reviewed in Carbonero et al. (2017)]. *VrREM1* is a homolog to *A. thaliana REM1* (*AtREM1*). Although *REM1* has not been reported to be associated with seed dormancy, a recent study showed that *REM1* is among 7 TFs possibly regulated the expression of 17 hub genes involved in dormancy transition of *Polygonatum kingianum* corm (Wang et al., 2020). *VrCML1* encodes a CML protein 1. Calmodulin (CaM) and CaM-like proteins are the major Ca^2+^-binding proteins, and their signaling has been shown to be involved in the ABA-induced inhibition of seed germination and seedling growth. In *A. thaliana*, transgenic lines with under-expressing *AtCML24* shows resistance to ABA-induced inhibition of seed germination and seedling growth (Delk et al., 2005). The expression of *AtCML9* is affected by ABA and abiotic stress, and the *cml9* null mutant shows a hypersensitive response to ABA during seed germination and seedling growth (Magnan et al., 2008). *A. thaliana* transgenic lines possessing a novel CML gene, *OsMSR2*, from rice (*Oryza sativa* L.) exhibited hypersensitivity to ABA during seed germination and post-germination growth (Xu et al., 2011). Recently, the genes that are homologous to calcium signaling pathway-related genes, including *CALMODULIN-BINDING RECEPTOR-LIKE CYTOPLASMIC KINASE 3, CALCIUM-BINDING PROTEIN, CALMODULIN-RELATED PROTEIN, CBL-INTERACTING PROTEIN KINASE 31, CALMODULIN-BINDING PROTEIN 60D-LIKE, and CALCIUM-DEPENDENT PROTEIN KINASE* were found to be related with seed dormancy in a wheat (*Triticum aestivum* L.) mutant (Rikiishi et al., 2021). In case, *VrREM1* and/or *VrCML1* are truly involved in the dormancy, it would indicate that both physical and physiological dormancy controls the dormancy in ACC41.

## Data Availability Statement

The original contributions presented in the study are included in the article/Supplementary Material, further inquiries can be directed to the corresponding authors.

## Author Contributions

PS and KL conceived the idea, designed the studies, and wrote and revised the manuscript. KL, KA, and TY carried out field experiments and phenotyping. KL, KA, JC, YL, XY, and LW conducted a DNA marker analysis, a gene expression analysis, and DNA sequencing. KL and KA conducted a microscopic observation. PS and XC secured research funding and coordinated the study. KL analyzed data. All authors approved the final version of the manuscript.

## Conflict of Interest

The authors declare that the research was conducted in the absence of any commercial or financial relationships that could be construed as a potential conflict of interest.

## Publisher’s Note

All claims expressed in this article are solely those of the authors and do not necessarily represent those of their affiliated organizations, or those of the publisher, the editors and the reviewers. Any product that may be evaluated in this article, or claim that may be made by its manufacturer, is not guaranteed or endorsed by the publisher.

## Footnotes

1
https://www.arabidopsis.org

2
https://phytozome-next.jgi.doe.gov
