# Supplementary material for: A Class II KNOX Gene, KNAT7-1, Regulates Physical Seed Dormancy in Mungbean [Vigna radiata (L.) Wilczek]
Source: Front Plant Sci. 2022 Mar 15;13:852373. doi: 10.3389/fpls.2022.852373 (PMC8965505; doi:10.3389/fpls.2022.852373)
Supplement: Supplementary file 2 [file Data_Sheet_1.PDF]

Supplementary Figure S1. Sequence alignment of upstream sequence of *LOC106767068* (*VrKNAT7*) in ACC41, Kamphaeng Saen 2 (KPS2) and VC1973A (reference genome). ACC41 is a wild mungbean and has high percentage of dormant seeds, while KPS2 and VC1973A are cultivated mungbean and have no dormant seeds. Regions highlighted in blue and yellow are 5' untranslated region and the first exon, respectively. Regions with possible role as TATA-boxes are identified and indicated by red and bolded letters.

```
ACC41          TTTCAGTAACAATATGTTATCTTATTCAATATTTCAATTCTTTTACATAAATTAGGATCA   60
KPS2           TTTCAGTAACAATATGTTATCTTATTCAATATTTCAATTCTTTTACATAAATTAGGATCG   60
VC1973A        TTTCAGTAACAATATGTTATCTTATTCAATATTTCAATTCTTTTACATAAATTAGGATCG   60
               ***********************************************************

ACC41          TATATATATATATATATATATATATATATATATATATATATATATATCTATATTTTT-   119
KPS2           TATATATATCTC---------------------------------------TATATTTTTA   82
VC1973A        TATATATATCTC---------------------------------------TATATTTTTA   82
               ********* *                                       *********

ACC41          AAAATAAATAAAAAATAGTCTGTTTAGACAAATTTTAAGAAAACACGTATATAACAAGAA   179
KPS2           AAATAAATAAAAAAATTGGTCTGTTTAGACAAATTTTAAGAAAACACGTATATAATAAGAA   142
VC1973A        AAATAAATAAAAAAATTGGTCTGTTTAGACAAATTTTAAGAAAACACGTATATAATAAGAA   142
               ***  **  ***** * ************************************* *****

ACC41          AACGAATGATAAACATATAAAATTATTTATAAATTAAAATTAACTATAAATGCATTAAAA   239
KPS2           AACGAATGATAAACATATAAAATTATTTATAAATTAAAATTAACTATAAATGCATTAAAA   202
VC1973A        AACGAATGATAAACATATAAAATTATTTATAAATTAAAATTAACTATAAATGCATTAAAA   202
               ************************************************************

ACC41          AATATATTTCTTTAACAAATAACATGCAGAAAAAATGAGAAAGCTTGTAAATTAATTAAT   299
KPS2           AATATATTTCTTTAACAAATAACATTCAGAAAAAATGAGAGAGCTTGTAAATTAATTAAT   262
VC1973A        AATATATTTCTTTAACAAATAACATTCAGAAAAAATGAGAGAGCTTGTAAATTAATTAAT   262
               ************************* ************** *******************

ACC41          TTATGCTTTAGTAATTTGTTATGAGAAGATTTTATTTATTTGTTTGAAAGTGTTTTGGAA   359
KPS2           TTATGCTTTAGTAATTTGTTATGAGAAGATTTTATTTATTTGTTTGAAAGTGTTTTGGAA   322
VC1973A        TTATGCTTTAGTAATTTGTTATGAGAAGATTTTATTTATTTGTTTGAAAGTGTTTTGGAA   322
               ************************************************************

ACC41          CAGAAATGTAAAAATGTAAACGAAATATGAAAATGAAGAATTATGCAATAAATAATTACA   419
KPS2           CAGAAATGTAAAAATGTAAACGAAATATGAAAATGAAGAATTATGCAATAAGTAATTACA   382
VC1973A        CAGAAATGTAAAAATGTAAACGAAATATGAAAATGAAGAATTATGCAATAAGTAATTACA   382
               *************************************************** ********

ACC41          TAATAAAAATTAGGATAATAATATTTTAATAATTTTTTTTATTAACTTTTTTA-ATATAA   478
KPS2           TAATAAAAATTAGCACAATGATATTTTAACAACTTTTTTTTAACAATTTTTTTATGACAG   442
VC1973A        TAATAAAAATTAGCACAATGATATTTTAACAACTTTTTTTTAACAATTTTTTTATGACAG   442
               ************* * *** ********* ** *******    * ******    * *

ACC41          AATATATGTCACTATTTTATTAGTT-TTTTTAATTTATTTTAAAAATATATTTAAAACAG   537
KPS2           GATATGTGTTGCCATTTTATTGATTTGATTAAATTTATTCTAAAAATATATTTAAAACAG   502
VC1973A        GATATGTGTTGCCATTTTATTGGTTTGATTAAATTTATTCTAAAAATATATTTAAAACAG   502
                **** ***  * ********  **   ** ******** ********************

ACC41          ATAAATCAATCACAAAGTGTTATGTATTATAA-----------AAAAAAAGTTATAAAAA   586
KPS2           ACCAATCACAAAGTGTGATATATGTATTGTAAAAAAAATTATGAAAAAAAGTTGTTAAAA   562
VC1973A        ACCAATCACAAAGTGTGATATATGTATTGTAAAAAAAATTATGAAAAAAAGTTGTTAAAA   562
               *  *****   *    *   ******** ***           ********** * ****

ACC41          AAAGTTGTTAAAAAAAAATTAATACCCCTTATCCGCACAAATGTTGATCGTTCGTTGCTTT   646
KPS2           GATAATTTTCCTAAAAATTAATACCCCTTATCCGCACAAATGTTGATCGTTGGTTGCTTT   622
VC1973A        GATAATTTTCCTAAAAATTAATACCCCTTATCCGCACAAATGTTGATCGTTGGTTGCTTT   622
                *   * **   *************************************** ********

ACC41          TTGTCCGTGGCGTCT--GTGTATGGAAACTCGTTGTTCACGTCTGAGAGAGAAATAGAGA   704
KPS2           TTGTCCGTGGCGTCTTGGTGTATGGAAACTCGTTGTTCACGTCTGAGAGAGAAATAGAGA   682
VC1973A        TTGTCCGTGGCGTCTTGGTGTATGGAAACTCGTTGTTCACGTCTGAGAGAGAAATAGAGA   682
```

```
                ***************  *******************************************
ACC41           GCGATGTAAGGGTTATAAAAAGAGTCGGAGAAGGAGAGATCGGAATTTACAGGTGTCAGT    764
KPS2            GCGATGTAAGGGTTATAAAAAGAGTCGGAGAAGGAGAGATCGGAATTTACAGGTGTCAGT    742
VC1973A         GCGATGTAAGGGTTATAAAAAGAGTCGGAGAAGGAGAGATCGGAATTTACAGGTGTCAGT    742
                ************************************************************

ACC41           GCTTCCATGCTAGCATTGCATCACCATTGCCATACATAGAGTGGTCCTCAACTCAACCTC    824
KPS2            GCTTCCATGCTAGCATTGCATCACCATTGCCATACATAGAGTGGTCCTCAACTCAACCTC    802
VC1973A         GCTTCCATGCTAGCATTGCATCACCATTGCCATACATAGAGTGGTCCTCAACTCAACCTC    802
                ************************************************************

ACC41           ATTCTTTTCCCTTAAATAATCTCTCCCTCTCTAATTCCCTAATCCCTATCTTAACCCCCT    884
KPS2            ATTCTTTTCCCTTAAATAATCTCTCCCTCTCTAATTCCCTAATCCCTATCTTAACCCCCT    862
VC1973A         ATTCTTTTCCCTTAAATAATCTCTCCCTCTCTAATTCCCTAATCCCTATCTTAACCCCCT    862
                ************************************************************

ACC41           TCTCTTCCCTTAATCACTGCAGATGCAAGAAGCTGGGCTGGCAATGAATATGCTCAGCGC    944
KPS2            TCTCTTCCCTTAAACACTGCAGATGCAAGAAGCTGGGCTGGCAATGAATATGCTCAGCGC    922
VC1973A         TCTCTTCCCTTAAACACTGCAGATGCAAGAAGCTGGGCTGGCAATGAATATGCTCAGCGC    922
                ************* **********************************************

ACC41           AGAAGTCTCCGCCGCCGCCGCCGA    968
KPS2            AGAAGTCTCCGCCGCCGCCGCCGA    946
VC1973A         AGAAGTCTCCGCCGCCGCCGCCGA    946
                ************************
```
