# Supplementary material for: A Class II KNOX Gene, KNAT7-1, Regulates Physical Seed Dormancy in Mungbean [Vigna radiata (L.) Wilczek]
Source: Front Plant Sci. 2022 Mar 15;13:852373. doi: 10.3389/fpls.2022.852373 (PMC8965505; doi:10.3389/fpls.2022.852373)
Supplement: Supplementary file 3 [file Data_Sheet_2.PDF]

Supplementary Figure S2. Sequence alignment of coding sequence of *VrKNAT7* gene from wild mungbean ACC41 and cultivated mungbeans Kamphaeng Saen 2 (KPS2) and VC1973A (reference sequence). Polymorphism site is bolded and highlighted in red.

```
ACC41        ATGCAAGAAGCTGGGCTGGCAATGAATATGCTCAGCGCAGAAGTCTCCGCCGCCGCCGCC    60
KPS2         ATGCAAGAAGCTGGGCTGGCAATGAATATGCTCAGCGCAGAAGTCTCCGCCGCCGCCGCC    60
VC1973A      ATGCAAGAAGCTGGGCTGGCAATGAATATGCTCAGCGCAGAAGTCTCCGCCGCCGCCGCC    60
             ************************************************************

ACC41        GACCACCACCACCGTCAACTGAAGGCGGACATAGCCACCCATCCACTTTACGAACAGCTT    120
KPS2         GACCACCACCACCGTCAACTGAAGGCGGACATAGCCACCCATCCACTTTACGAACAGCTT    120
VC1973A      GACCACCACCACCGTCAACTGAAGGCGGACATAGCCACCCATCCACTTTACGAACAGCTT    120
             ************************************************************

ACC41        CTCGCTGCACACGTCTCATGCCTCCGCGTGGCTACACCGATCGACCAGTTGCCACTGATC    180
KPS2         CTCGCTGCACACGTCTCATGCCTCCGCGTGGCTACACCGATCGACCAGTTGCCACTGATC    180
VC1973A      CTCGCTGCACACGTCTCATGCCTCCGCGTGGCTACACCGATCGACCAGTTGCCACTGATC    180
             ************************************************************

ACC41        GACGCTCAGTTATCACACTTCAACAATCTCCTCCGCTCTTACGCCTCACACCATTCCCAT    240
KPS2         GACGCTCAGTTATCACACTTCAACAATCTCCTCCGCTCTTACGCCTCACACCATTCCCAT    240
VC1973A      GACGCTCAGTTATCACACTTCAACAATCTCCTCCGCTCTTACGCCTCACACCATTCCCAT    240
             ************************************************************

ACC41        TCCCATTCCCATGATCGACAAGAGCTCGACAACTTCATGACACAGTATTTGATCGTGTTA    300
KPS2         TCCCATTCCCATGATCGACAAGAGCTCGACAACTTCATGACACAGTATTTGATCGTGTTA    300
VC1973A      TCCCATTCCCATGATCGACAAGAGCTCGACAACTTCATGACACAGTATTTGATCGTGTTA    300
             ************************************************************

ACC41        TGTGCGTTAAAAGAGCAGCTTCAGCAACATGTTCGGGTGCATGCCGTGGAGGCTGTGATG    360
KPS2         TGTGCGTTAAAAGAGCAGCTTCAGCAACATGTTCGGGTGCATGCCGTGGAGGCTGTGATG    360
VC1973A      TGTGCGTTAAAAGAGCAGCTTCAGCAACATGTTCGGGTGCATGCCGTGGAGGCTGTGATG    360
             ************************************************************

ACC41        GCCTGCCGTGATATTGAAAGCACCTTGCAAGCTCTGACAGGAGTGAGCCTGGGAGAAGGA    420
KPS2         GCCTGCCGTGATATTGAAAGCACCTTGCAAGCTCTGACAGGAGTGAGCCTGGGAGAAGGA    420
VC1973A      GCCTGCCGTGATATTGAAAGCACCTTGCAAGCTCTGACAGGAGTGAGCCTGGGAGAAGGA    420
             ************************************************************

ACC41        TCGGGTGCAACAATGTCAGATGACGAAGAGGATTTTCAAATGGATGGGTCTTTAGATCAG    480
KPS2         TCGGGTGCAACAATGTCAGATGACGAAGAGGATTTTCAAATGGATGGGTCTTTAGATCAG    480
VC1973A      TCGGGTGCAACAATGTCAGATGACGAAGAGGATTTTCAAATGGATGGGTCTTTAGATCAG    480
             ************************************************************

ACC41        TCTAGCGCTGAGGGGCACGACATGATGGGATTTGGTCCATTGCTTCCTACAGAATCTGAA    540
KPS2         TCTAGCGCTGAGGGGCACGACATGATGGGATTTGGTCCATTGCTTCCTACAGAATCTGAA    540
VC1973A      TCTAGCGCTGAGGGGCACGACATGATGGGATTTGGTCCATTGCTTCCTACAGAATCTGAA    540
             ************************************************************

ACC41        AGGTCCCTCATGGAAAGAGTTCGTCAGGAGCTCAAAATTGAGCTCAAGCAGGGTTTCAAG    600
KPS2         AGGTCCCTCATGGAAAGAGTTCGTCAGGAGCTCAAAATTGAGCTCAAGCAGGGTTTCAAG    600
VC1973A      AGGTCCCTCATGGAAAGAGTTCGTCAGGAGCTCAAAATTGAGCTCAAGCAGGGTTTCAAG    600
             ************************************************************

ACC41        TCAAGAATCGAAGATGTTAGGGAGGAAATATTAAGGAAAAGGAGAGCCGGGAAATTGCCT    660
KPS2         TCAAGAATCGAAGATGTTAGGGAGGAAATATTAAGGAAAAGGAGAGCCGGGAAATTGCCT    660
VC1973A      TCAAGAATCGAAGATGTTAGGGAGGAAATATTAAGGAAAAGGAGAGCCGGGAAATTGCCT    660
             ************************************************************

ACC41        GGGGACACAACATCAGTGTTAAAGGCATGGTGGCAGCAACACGCTAAATGGCCCTACCCA    720
KPS2         GGGGACACAACATCAGTGTTAAAGGCATGGTGGCAGCAACACGCTAAATGGCCCTACCCA    720
VC1973A      GGGGACACAACATCAGTGTTAAAGGCATGGTGGCAGCAACACGCTAAATGGCCCTACCCA    720
             ************************************************************

ACC41        ACTGAAGATGACAAGGCAAAACTTGTGGAAGAGACAGGGTTGCAGCTGAAGCAAATCAAT    780
KPS2         ACTGAAGATGACAAGGCAAAACTTGTGGAAGAGACAGGGTTGCAGCTGAAGCAAATCAAT    780
VC1973A      ACTGAAGATGACAAGGCAAAACTTGTGGAAGAGACAGGGTTGCAGCTGAAGCAAATCAAT    780
```

```
               ************************************************************

ACC41          AATTGGTTCATCAACCAAAGGAAACGCAACTGGCACAGCAACTCTCAATCGGTCACCTCT   840
KPS2           AATTGGTTCATCAACCAAAGGAAACGCAACTGGCACAGCAACTCTCAATCGGTCACCTCT   840
VC1973A        AATTGGTTCATCAACCAAAGGAAACGCAACTGGCACAGCAACTCTCAATCGGTCACCTCT   840
               ************************************************************

ACC41          CTCAAGTCCAAGCGTAAGAGGGAGTATTCCCACTAA  876
KPS2           CTCAAGTCCAAGCGCAAGAGGGAGTATTCCCACTAA  876
VC1973A        CTCAAGTCCAAGCGCAAGAGGGAGTATTCCCACTAA  876
               ************** *********************
```
