# Supplementary material for: A Class II KNOX Gene, KNAT7-1, Regulates Physical Seed Dormancy in Mungbean [Vigna radiata (L.) Wilczek]
Source: Front Plant Sci. 2022 Mar 15;13:852373. doi: 10.3389/fpls.2022.852373 (PMC8965505; doi:10.3389/fpls.2022.852373)
Supplement: Supplementary file 4 [file Data_Sheet_3.PDF]

Supplementary Figure S3. Sequence alignment of VrKNAT7 proteins from wild mungbean ACC41 and cultivated mungbeans Kamphaeng Saen 2 (KPS2) and VC1973A (reference sequence).

```
ACC41        MQEAGLAMNMLSAEVSAAAADHHHRQLKADIATHPLYEQLLAAHVSCLRVATPIDQLPLI    60
KPS2         MQEAGLAMNMLSAEVSAAAADHHHRQLKADIATHPLYEQLLAAHVSCLRVATPIDQLPLI    60
VC1973A      MQEAGLAMNMLSAEVSAAAADHHHRQLKADIATHPLYEQLLAAHVSCLRVATPIDQLPLI    60
             ************************************************************

ACC41        DAQLSHFNNLLRSYASHHSHSHSHDRQELDNFMTQYLIVLCALKEQLQQHVRVHAVEAVM    120
KPS2         DAQLSHFNNLLRSYASHHSHSHSHDRQELDNFMTQYLIVLCALKEQLQQHVRVHAVEAVM    120
VC1973A      DAQLSHFNNLLRSYASHHSHSHSHDRQELDNFMTQYLIVLCALKEQLQQHVRVHAVEAVM    120
             ************************************************************

ACC41        ACRDIESTLQALTGVSLGEGSGATMSDDEEDFQMDGSLDQSSAEGHDMMGFGPLLPTESE    180
KPS2         ACRDIESTLQALTGVSLGEGSGATMSDDEEDFQMDGSLDQSSAEGHDMMGFGPLLPTESE    180
VC1973A      ACRDIESTLQALTGVSLGEGSGATMSDDEEDFQMDGSLDQSSAEGHDMMGFGPLLPTESE    180
             ************************************************************

ACC41        RSLMERVRQELKIELKQGFKSRIEDVREEILRKRRAGKLPGDTTSVLKAWWQQHAKWPYP    240
KPS2         RSLMERVRQELKIELKQGFKSRIEDVREEILRKRRAGKLPGDTTSVLKAWWQQHAKWPYP    240
VC1973A      RSLMERVRQELKIELKQGFKSRIEDVREEILRKRRAGKLPGDTTSVLKAWWQQHAKWPYP    240
             ************************************************************

ACC41        TEDDKAKLVEETGLQLKQINNWFINQRKRNWHSNSQSVTSLKSKRKREYSH    291
KPS2         TEDDKAKLVEETGLQLKQINNWFINQRKRNWHSNSQSVTSLKSKRKREYSH    291
VC1973A      TEDDKAKLVEETGLQLKQINNWFINQRKRNWHSNSQSVTSLKSKRKREYSH    291
             ***************************************************
```
