# Supplementary material for: A Class II KNOX Gene, KNAT7-1, Regulates Physical Seed Dormancy in Mungbean [Vigna radiata (L.) Wilczek]
Source: Front Plant Sci. 2022 Mar 15;13:852373. doi: 10.3389/fpls.2022.852373 (PMC8965505; doi:10.3389/fpls.2022.852373)
Supplement: Supplementary file 5 [file Data_Sheet_4.PDF]

Supplementary Table S2. Location and effect of QTL *Sdwa5.1.1+* on linkage group 2 controlling seed dormancy detected in mungbean $F_{2:3}$ population of 100 individuals of the cross Kamphaeng Saen 2 (KPS2) × ACC41

LOD score	Position on linkage group (cM)	Marker interval	Phenotypic variance explained (%)	Additive effect	Dominant effect
7.75	1.90	VrSdp-SSR5 – VrKNAT7-SSR4	31.75	-18.42	11.85
