# Supplementary material for: A Class II KNOX Gene, KNAT7-1, Regulates Physical Seed Dormancy in Mungbean [Vigna radiata (L.) Wilczek]
Source: Front Plant Sci. 2022 Mar 15;13:852373. doi: 10.3389/fpls.2022.852373 (PMC8965505; doi:10.3389/fpls.2022.852373)
Supplement: Supplementary file 6 [file Data_Sheet_5.PDF]

Supplementary Table S3. Details of annotated genes locating between markers VrSpd-SSR102 and VrSpd-SSR104 flanking the QTL *Sdwa5.1.1+* controlling seed dormancy.

**NCBI annotated genes**	**Location on mungbean reference genome (Kang et al. 2014) (chromosome:position)**	**Function**
LOC106768002	7:27,448,720..27,453,102	B3 domain-containing protein Os01g0234100
LOC106767865	7:27,454,224..27,455,687	Uncharacterized protein
LOC106767275	7:27,467,893..27,468,682	Calmodulin-like protein 1
LOC106766251	7:27,476,196..27,478,195	Protein DETOXIFICATION 49-like
LOC111241965	7:27,487,079..27,487,354	60S ribosomal protein L29-1-like
